# Insights into the genetics of body size in the Bull Terrier

**DOI:** 10.1111/age.70000

**Published:** 2025-01-28

**Authors:** Claire M. Wade

**Affiliations:** ^1^ School of Life and Environmental Sciences University of Sydney Sydney New South Wales Australia

**Keywords:** body‐size, dog, gene, SNP, variant

## Abstract

The Bull Terrier (Miniature) and Bull Terrier are two varieties of a dog breed historically divided by size. We identify variety‐associated chromosomal regions identified using stratified genome‐wide association analysis of 69 Bull Terriers (Miniature) and 33 Bull Terriers. Next, we assess the significance of possible functional variants for body size using height (*N* = 1458) and weight (*N* = 1282) of Dog10K individuals with breed‐representative metrics available. Variants significant for size across breeds that are consistent with size alleles observed in four Bull Terriers and four Bull Terriers (Miniature) represented in Dog10K are highlighted. From five identified regions, two include genes already known to influence canine body size and a third contains a potential new height gene (ARFGEF3). Near *LCORL*, the most highly associated variant for height in Bull Terriers was chr3:91734656A>G (*p*_Across‐breed height_ = 2.459 × 10^−99^) and for weight it was chr3:91706639G>A (*p*_Across‐breed weight_ = 9.762 × 10^−85^). All Bull Terriers (including Miniature) were monomorphic for the derived allele at the known size variant in *LCORL* (chr3:91872822A>del). In the first exon of *IGF2BP2*, the derived allele at chr34:18694869‐71ins>del significantly reduces both height and weight in Bull Terriers and across breeds (Dog10K breed representative height and weight) (*p*_Across‐breed height_ = 1.65 × 10^−9^; *p*_Across‐breed weight_ = 1.79 × 10^−8^). The derived allele of the missense variant in *ARFGEF3* chr1:30793904G>A, XP_038382065.1 p.V243I significantly reduces breed representative height but not weight (*p*_Across‐breed height_ = 0.01). The effects on the variants assessed are limited to small variants identified in the Dog10K resource using breed‐representative sizes.

Most domestic dog breeds have size requirements (weight and height) that are specified by breed standards that also include descriptions of other physical and behavioural traits that typify animals within the breed. While many genes contributing to size differences among domestic dog breeds have been proposed and validated (Plassais et al., 2019; Raymond et al., 2022; Rimbault et al., 2013), it is well understood that the traits exhibit complex inheritance. Genes and specific variants generating size differences between varieties of single breeds remain to be explicitly described for most dog breeds.

The Bull Terrier is stratified by size into the Bull Terrier (BT) and Bull Terrier (Miniature) (MBT) varieties. The Bull Terrier dog breed was developed in England during the nineteenth century (The Kennel Club, 2009b). The BT breed standard specifies no weight or height limits other than that individuals should demonstrate maximum substance for their frame and sex (The Kennel Club, 2009b). Despite this, most BT weigh between 23 and 32 kg and stand 53–56 cm at the wither.

The MBT was first recognised as a separate variety from the BT in 1943 (The Kennel Club, 2009a) and was reportedly established through the direct selection of smaller BTs. According to the Kennel Club breed standard, the preferred size for the MBT is smaller than 35.5 cm (14 inches) (The Kennel Club, 2009a). The MBT weighs ~15 kg. Restricted interbreeding between the two varieties is allowed (The Kennel Club, 2009a, 2009b). The resulting puppies are registered as MBT.

Using public data, this study applies genome‐wide association analysis (GWAS) to identify known and potentially new canine body size genes in genomic regions that are diverged between the two varieties of Bull Terrier. The BT and MBT variant calls from dogs with whole genome sequences are then used to identify possible functional genetic variants at associated candidate body size loci.

No samples from new animals were used in this study and all data are in the public domain. Illumina canine genotyping array data (220853 SNPs) were available from 72 BT and 92 MBT genotyped for two previous studies investigating lethal acrodermatitis (All *N* = 78, BT *N* = 72, MBT *N* = 6) and laryngeal paralysis (All MBT *N* = 86) (Bauer et al., 2018; Hadji Rasouliha et al., 2019). Metadata describing breed variety were available with the data.

Animals were assigned unambiguously to their breed variety using multidimensional scaling of genotyping array data with plink (Chang et al., 2015). Inter‐variety case–control GWAS was then conducted using the BT as control and MBT as case. Acceptance of associated genome regions for further consideration required three co‐located markers associated by chi‐squared probability *p*_33BT_v_69MBT_ < 1 × 10^−15^.

The Dog10K consortium data resource (Dog10K) variant call file resource was used for mutation detection in associated regions (Meadows et al., 2023). It includes 1987 individual canine and canid records including BT (*N* = 4) and MBT (*N* = 4). All known variants within accepted regions were captured. Representative BT and MBT were used to identify potential breed‐variety associated functional variants in the accepted regions (Table 1).

**TABLE 1:** Variant characteristics of five chromosomal regions with more than three markers associated at *p* < 1 × 10^−15^ in inter‐variety genome‐wide association analysis.

Chromosome (chr)	Region (UU_Cfam_GSD1.0)	Strongly significant array markers (*N*)	Local variety‐polymorphic VCF variants (*N*)^a^	Local variety‐associated VCF variants (*N*)^b^	Local candidate genes
1	chr1:29753955–31196247	9	4952	1081	*ARFGEF3*
3	chr3:90323433–92234203	25	6812	3397	*LCORL*
10	chr10:22419486–27856291	72	16 302	445	n/a
18	chr18:30893366–37177480	23	26 624	1342	n/a
34	chr34:18646348–18939475	4^c^	651	448	*IGF2BP2*
Total		132	55 341	6713	

^a^ Regional VCF (variant call file) variants from Meadows et al. (2023) polymorphic in four BT and four MBT.

^b^ Regional VCF variants associated with *p* < 0.01 in four BT and four MBT representatives.

^c^ One marker unavailable in UU_Cfam_GSD1.0 reference.

Where available, breed‐mean phenotypes for height (cm) and weight (kg) were assigned to the Dog10K data and local across‐breed quantitative trait associations were conducted for variants showing inter‐variety association *p*_4BT_v_4MBT_ < 0.01 to assess the possible functional consequences of the BT vs. MBT associated variants across breeds. The allelic direction of weight or height gain across breeds was quantified and assessed for agreement BT and MBT breed alleles. Full methods can be found in Appendix S1.

Multidimensional scaling identified the main clusters and individuals strongly concordant with the BT and MBT varieties (Figure S1, Table S1). The analysis retained 69 MBT (case) and 33 BT (control) for the inter‐variety GWAS. No attempt was made to address stratification (lambda_median Chi‐squared_ = 10.778), as size is the primary reason for varietal segregation. Bonferroni significance identified 5294 markers with *p*_BT‐MBT_ ≤ 5.4 × 10^−7^ (Table S2); however, the arbitrary truncation point for acceptance in further analysis was set as *p*_BT‐MBT_ ≤ 1 × 10^−15^.

Five genomic regions met inter‐variety association criteria on chromosomes (chr) 1, 3, 10, 18 and 34 (Appendix S1, Table 1, Table S2, Figure S2). Two identified regions include known canine size genes: *LCORL* (*Ligand Dependent Nuclear Receptor Corepressor Like*) on chr 3, and *IGF2BP2* (*Insulin Like Growth Factor 2 MRNA Binding Protein 2*) on chr 34 (Figure 1). Within the five regions, 6713 variants from Dog10K data segregated with variety size in the four BT and four MBT representatives with *p* < 0.01 (Table 1, Table S3). Quantitative across‐breed association scores for breed mean heights and weights, restricted to 55 341 BT–MBT variants with minor allele frequency >0.01 in BT and MBT are reported (Table S3), as are genotype mean height and weight across breeds for 54 variants in transcribed sequences (Table S4).

- *LCORL* – body height and weight are complex traits, so multiple functional variants may exist within known body size genes. The BT and MBT assessed in this study were all homozygous for the deletion allele (smaller body size) of the known variant in *LCORL* described previously (Plassais et al., 2019), yet still demonstrated strong inter‐variety association at *LCORL*. Near *LCORL*, the most highly associated inter‐variety height variant was chr3:91734656A>G (*p*_4BT_v_4MBT_ = 0.007; *p*_Across‐breed height_ = 2.459 × 10^−99^) and for weight was chr3: 91706639G>A (*p*_4BT_v_4MBT_ = 0.007; *p*_Across‐breed weight_ = 9.762 × 10^−85^). Both variants map to transcribed sequences (RLOC_0020773.1 and LOC119871273) (Tables S2 and S4, Figure 1).
- *IGF2BP2* – putative functional variants for canine body size at *IGF2BP2* are yet to be described (Jones et al., 2008; Plassais et al., 2019). For BT v. MBT, both height and weight were associated with an insertion–deletion variant UU_Cfam_GSD_1.0 (GCF_011100685.1) 18694869‐71CTC>del also significant across breeds (*p*_4BT_v_4MBT_ = 0.002; *p*_Across‐breed height_ = 1.65 × 10^−9^; *p*_Across‐breed weight_ = 1.79 × 10^−8^). The variant results in a potential in‐frame deletion of a glutamine in the first exon of IGF2BP2. Across breeds, the variant reduces mean height and weight in a dominant/recessive manner: height – del/del 44 cm, del/ins 44 cm, ins/ins 50 cm; and weight – del/del 17 kg, del/ins 18 kg, ins/ins 24 kg (Tables S2 and S4, Figure 1).

**FIGURE 1:**
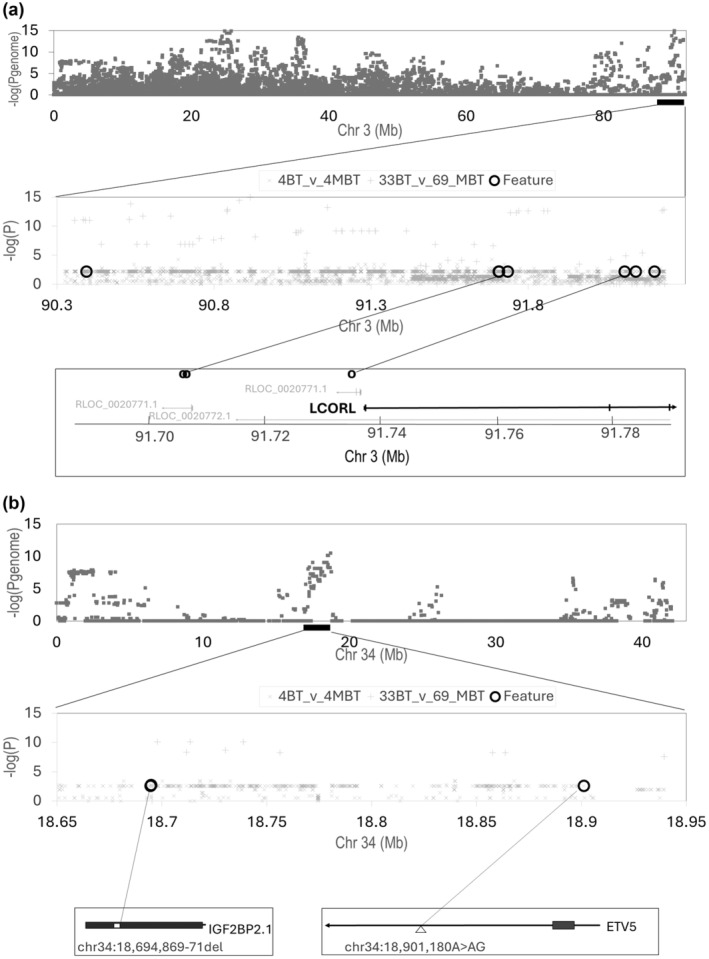
Chromosome and regional associations (−log *p*_raw_4BT_v_4MBT_, −log *p*_genome 33BT_v_69MBT_) for inter‐variety difference between Bull Terrier and Bull Terrier (Miniature) in the vicinities of two known canine size genes. (a) *LCORL* region on chromosome 3; (b) *IGF2BP2* region on chromosome 34.

Other chromosomal regions identified in this analysis contain no known canine body size genes; however on chr 1, a missense variant in ARFGEF3 (*ARFGEF Family Member 3*), XP_038382065.1 p.V243I (*p*_4BT_v_4MBT_ = 0.0023; *p*_Across‐breed___height_ = 0.01, *p*_Across‐breed___weight_ = 0.74) is associated with a potential 10 cm shift in height across breeds that is likely related to insulin signalling. Full results for the five loci and further discussion can be found in Appendix S2.

We conclude that body size differences between BT and MBT can be at least partially explained by variants that differ from those already described in the canine literature at two known size loci, *LCORL* and *IGF2BP2*. The insulin signalling gene *ARFGEF3* is also predicted to influence height difference between the two breeds. For dog breeds where small body size is desirable, identification of size‐reducing variants without negative welfare consequences may prove valuable to attain reduced body sizes in breeding programmes.

## CONFLICT OF INTEREST STATEMENT

The author declares no conflict of interest.

## Supporting information


Appendix S1.

Appendix S2.

Figure S1.

Figure S2.

Table S1.
Table S2.
Table S3.
Table S4.

## Data Availability

All data used in this analysis are available in the public domain via references provided in the text.
