# Supplementary material for: Insights into the genetics of body size in the Bull Terrier
Source: Anim Genet. 2025 Jan 28;56(1):e70000. doi: 10.1111/age.70000 (PMC11773297; doi:10.1111/age.70000)
Supplement: Supplementary file 1 — Appendix S1. [file AGE-56-0-s004.docx]

## Appendix 1. Supplemental Methods

### Assignment of individuals to Bull Terrier breed-variety

Data were analyzed using the PLINK version 1.9 genetic analysis toolkit (Chang et al. 2015). Data were quality filtered for individual dog call rate (--mind 0.1), individual marker calling rate (--geno 0.1), marker adherence to Hardy-Weinberg expectation (--hwe 0.0005) and minor-allele frequency (--maf 0.05).

Multi-dimensional scaling (--cluster --mds-plot 2) was used to visualise the spatial dispersion of animals identified as MBT and BT. Individuals that did not cluster with the main group of others identified as the same variety (according to descriptions in previous data resource meta-data) were excluded from association analysis. This included some dogs with coordinate locations between the BT and MBT main clusters that may have resulted from sanctioned inter-varietal breeding. Arbitrary criteria for exclusion were C1<-0.04 for BT and C1>-0.01 for MBT.

### Genome-wide association analysis (GWAS)

Genome-wide association for breed variety was conducted (MBT as case and BT as control). This allowed the population stratification to be quantified. The acceptable level for association significance based on Bonferroni (--assoc --adjust) was ascertained. The same quality filtering was applied for GWAS as for the multi-dimensional scaling analysis.

### Identification of chromosomal regions of interest

Acceptance of associated regions for further consideration required that chromosomal regions have at least three markers associated by Chi-squared probability P_69MBT_v_33BT_< 1e^-15^ with breed variety and that co-located markers must have inter marker distances of less than one megabase (Mb) (Karlsson et al. 2007).

The beginning and end of each region of interest was specified by the base location of the genotyped array variant immediately before the first, and after the last strongly significant marker (P_69MBT_v_33BT_ <1e^-15^) in each cluster of associated markers. Only variants and individuals meeting quality filtering criteria (--mind 0.1, --geno 0.1, --maf 0.01) were considered for this purpose.

### Across-breed validation of size variation within regions of interest

Within the regions of interest identified using the inter-variety GWAS, all known variants were captured from the Dog10K consortium data resource (Dog10K) (Meadows et al. 2023) using the TABIX tool (Li 2011). The Dog10K resource consists of two curated variant call files (VCF) representing 1,987 individual sequenced dog and canid genomes. One file contains single nucleotide (SNP) variants, and a second file contains non-SNP variants. The resource includes four individuals annotated as MBT, and four individuals annotated as BT that were used for local inter-variety association within regions identified in Analysis 1. Variant positions in the resource are reported relative to the UU_Cfam_GSD1.0 reference genome, commonly referred to as canFam4 (Wang et al. 2021). Where necessary, the LIFTOVER tool on the University of California (Santa Cruz) genome browser (UCSC browser) (Kent et al. 2002) was used to relocate array markers and regions between canine reference assemblies. Command-line resources were used to merge the SNP and non-SNP VCF data.

*Local case-control association*

Minor allele frequencies were assessed for 4 BT and 4 MBT in the Dog10K data using PLINK (Chang *et al.* 2015). Only variants polymorphic in the combined BT-MBT representative group and meeting general quality filtering criteria (--mind 0.1, --geno 0.1, --maf 0.01) were considered further. We refer to variants with non-zero minor allele frequency in BT and MBT as local variety-polymorphic variants. These variants were used to provide high resolution visibility of variation segregating with the regions observed as associated in the BT v. MBT array-based GWAS.

*Local across-breed quantitative association analyses*

Where available, breed-mean phenotypes for height (cm) and weight (kg) were assigned to individuals in the Dog10K VCF. The assigned values were taken from published analyses (Plassais et al. 2019), or from breed standards reported by major cynological registries including the American Kennel Club (AKC), The Kennel Club (KC) of the United Kingdom, Dogs Australia (AUS) or the Fédération Cynologique Internationale (FCI) (Table S1_BreedMeans). Size and weight data were collated for a University of Sydney honours thesis (Venerussi 2024). Animals with breed-mean height (N= 1,458) and weight (N= 1,282) breed-mean phenotypes were available (Table S5).

The analysis package PLINK (Chang et al. 2015) was used to conduct quantitative association of breed-mean height and weight for the subset of local variety-polymorphic variants. Only variants and individuals meeting quality filtering criteria (--mind 0.1, --geno 0.1, --maf 0.01) were considered. Missing height or weight values were coded as -9.

The variants with the strongest associations within each region were noted and the genotype means: height (cm) and weight (kg) were reported using the qt-means function in PLINK. The direction of allelic association with size was checked for concordance with expected size difference between the BT and MBT breed varieties.

Putative function was assigned to the locally polymorphic variants using the Variant Annotation Integrator tool within the UCSC browser (Hinrichs et al. 2016). Local variety-polymorphic variants were uploaded as the query track and Transcripts: NCBI RefSeq genes, curated and predicted (NM_*, XM_*, NR_*, XR_*, NP_*, YP_*) used as the comparators. Displayed variants were limited to those annotated as present in transcripts including untranslated regions, splice junctions and exons. In addition, manual curation was conducted for previously described size genes relative to transcripts annotated by the University of Uppsala for the UU_Cfam_GSD1.0 reference genome.

Attribution of functional effect on body size by genes within regions of interest was based on two major methods. First, previous nomination as a gene affecting canine body size according to the canine genetics literature (Raymond et al. 2022). Second, each gene was located on the Jackson Laboratories Mouse Phenome Database (Bogue et al. 2020). Where genes had nominated phenotypes for the phenotypic classes of “Growth”, “Craniofacial”, “Limb”, or “Skeleton”, the phenotypes were examined and any genes with phenotypes relevant to body size, weight, length, or substance were prioritized for further analysis. Where no mouse annotation was available, phenotypic information was obtained via Genecards (Stelzer et al. 2016).

For variants outside of transcripts, the variants were assessed by LIFTOVER to the hg38 human reference genome and manually observed relative to the regulatory annotation tracks Oreganno (Lesurf et al. 2016) and PhyloP (Pollard et al. 2010).

## References

1. Bogue M.A., Philip V.M., Walton D.O., Grubb S.C., Dunn M.H., Kolishovski G., Emerson J., Mukherjee G., Stearns T., He H., Sinha V., Kadakkuzha B., Kunde-Ramamoorthy G. & Chesler E.J. (2020) Mouse Phenome Database: a data repository and analysis suite for curated primary mouse phenotype data. Nucleic Acids Res 48, D716-D23.

2. Chang C.C., Chow C.C., Tellier L.C., Vattikuti S., Purcell S.M. & Lee J.J. (2015) Second-generation PLINK: rising to the challenge of larger and richer datasets. Gigascience 4, 7.

3. Hinrichs A.S., Raney B.J., Speir M.L., Rhead B., Casper J., Karolchik D., Kuhn R.M., Rosenbloom K.R., Zweig A.S., Haussler D. & Kent W.J. (2016) UCSC Data Integrator and Variant Annotation Integrator. Bioinformatics 32, 1430-2.

4. Karlsson E.K., Baranowska I., Wade C.M., Salmon Hillbertz N.H., Zody M.C., Anderson N., Biagi T.M., Patterson N., Pielberg G.R., Kulbokas E.J., 3rd, Comstock K.E., Keller E.T., Mesirov J.P., von Euler H., Kampe O., Hedhammar A., Lander E.S., Andersson G., Andersson L. & Lindblad-Toh K. (2007) Efficient mapping of mendelian traits in dogs through genome-wide association. Nat Genet 39, 1321-8.

5. Kent W.J., Sugnet C.W., Furey T.S., Roskin K.M., Pringle T.H., Zahler A.M. & Haussler D. (2002) The human genome browser at UCSC. Genome Res 12, 996-1006.

6. Lesurf R., Cotto K.C., Wang G., Griffith M., Kasaian K., Jones S.J., Montgomery S.B., Griffith O.L. & Open Regulatory Annotation C. (2016) ORegAnno 3.0: a community-driven resource for curated regulatory annotation. Nucleic Acids Res 44, D126-32.

7. Li H. (2011) Tabix: fast retrieval of sequence features from generic TAB-delimited files. Bioinformatics 27, 718-9.

8. Meadows J.R.S., Kidd J.M., Wang G.D., Parker H.G., Schall P.Z., Bianchi M., Christmas M.J., Bougiouri K., Buckley R.M., Hitte C., Nguyen A.K., Wang C., Jagannathan V., Niskanen J.E., Frantz L.A.F., Arumilli M., Hundi S., Lindblad-Toh K., Ginja C., Agustina K.K., Andre C., Boyko A.R., Davis B.W., Drogemuller M., Feng X.Y., Gkagkavouzis K., Iliopoulos G., Harris A.C., Hytonen M.K., Kalthoff D.C., Liu Y.H., Lymberakis P., Poulakakis N., Pires A.E., Racimo F., Ramos-Almodovar F., Savolainen P., Venetsani S., Tammen I., Triantafyllidis A., vonHoldt B., Wayne R.K., Larson G., Nicholas F.W., Lohi H., Leeb T., Zhang Y.P. & Ostrander E.A. (2023) Genome sequencing of 2000 canids by the Dog10K consortium advances the understanding of demography, genome function and architecture. Genome Biol 24, 187.

9. Plassais J., Kim J., Davis B.W., Karyadi D.M., Hogan A.N., Harris A.C., Decker B., Parker H.G. & Ostrander E.A. (2019) Whole genome sequencing of canids reveals genomic regions under selection and variants influencing morphology. Nat Commun 10, 1489.

10. Pollard K.S., Hubisz M.J., Rosenbloom K.R. & Siepel A. (2010) Detection of nonneutral substitution rates on mammalian phylogenies. Genome Res 20, 110-21.

11. Raymond P.W., Velie B.D. & Wade C.M. (2022) Forensic DNA phenotyping: Canis familiaris breed classification and skeletal phenotype prediction using functionally significant skeletal SNPs and indels. Anim Genet 53, 247-63.

12. Stelzer G., Rosen N., Plaschkes I., Zimmerman S., Twik M., Fishilevich S., Stein T.I., Nudel R., Lieder I., Mazor Y., Kaplan S., Dahary D., Warshawsky D., Guan-Golan Y., Kohn A., Rappaport N., Safran M. & Lancet D. (2016) The GeneCards Suite: From Gene Data Mining to Disease Genome Sequence Analyses. Curr Protoc Bioinformatics 54, 1 30 1-1 3.

13. Venerussi C. (2024) Undergraduate research:Identification of significant functional size variants in the IGF2BP2 gene influencing the size stratification of the Miniature and Standard Bull Terrier. In: *School of Life and Environmental Sciences*. The University of Sydney.

14. Wang C., Wallerman O., Arendt M.L., Sundstrom E., Karlsson A., Nordin J., Makelainen S., Pielberg G.R., Hanson J., Ohlsson A., Saellstrom S., Ronnberg H., Ljungvall I., Haggstrom J., Bergstrom T.F., Hedhammar A., Meadows J.R.S. & Lindblad-Toh K. (2021) A novel canine reference genome resolves genomic architecture and uncovers transcript complexity. Commun Biol 4, 185.
