# Supplementary material for: Insights into the genetics of body size in the Bull Terrier
Source: Anim Genet. 2025 Jan 28;56(1):e70000. doi: 10.1111/age.70000 (PMC11773297; doi:10.1111/age.70000)
Supplement: Supplementary file 2 — Appendix S2. [file AGE-56-0-s003.docx]

## File_S2_Supplemental Results and Discussion

#### Assignment of individuals to variety

We understand that breed-mean assumptions can be problematic and that not all individuals may comply with expectations according to breed standards. The separation of the two varieties using multi-dimensional scaling was intended to capture groups of individuals most alike within the variety cohort.

The data from animals that were not strongly clustered with the main varietal groups (N=31, 13 BT and 19 MBT), outliers from both MBT and BT (N=5, all described as BT), potentially mis-classified as the wrong variety (N=1, BT), or that failed quality filtering (N=24, 20 BT and 4 MBT) were excluded from the analysis (Figure S1, Table S2). This resulted in the removal of 39 BT and 23 MBT (33 BT and 69 MBT were retained). The five outlier animals (LAD_36, LAD_43, LAD_21, LAD_13 and LAD_28) were all described as Bull Terrier in Bauer et al (2018), further provenance of those samples is unknown, but they may represent crossbred animals with external appearance matching BT.

While only 4BT and 4MBT were represented in the Dog10K data, the animals demonstrated the same major allele effects at the known array-marker loci in identified regions as the wider cohort and can therefore regarded as representative of their variety. At most loci identified as associated in the main analysis, the allelic separation between the two variety cohorts in the array data was extremely strong. This provides confidence that the regions identified in this analysis enable high probability identification of the variety (BT or MBT) of any individual from these populations. At all loci, one or the other variety demonstrates a haplotype identified by the local 4BT_v_4MBT association signal that is close to fixation and that is expected to include the likely functional variant(s) separating the breed varieties (Table S3).

#### Chromosome 1

#### Within the chr1 associated region (chr1:29,753,955-31,196,247), one array variant was very strongly associated with BT breed-variety in the array data (chr1: 30,979,525, P_69MBT_V_33BT_=1.68e^-21^), however the remaining eight passing array markers were closer to the lower significance cut-off for further analysis (probabilities between P_69MBT_V_33BT_ =1.2e^-16^ and 9.624e^-16^) (Table S3). The chr 1 region contains no previously reported canine size genes (Raymond et al. 2022).

The most strongly associated inter-varietal array variant in the chr 1 region (chr1: 30,977,883C>T) is also a missense variant in the gene NHS-Like Protein 1 (XP_038382074.1: p.A1,313S, manually curated). While NHSL1 has no obvious connection with skeletal body-size, it is curated as being involved in the production of thyroid hormones and their downstream signalling (Stelzer et al. 2016). Thyroid hormones are known to affect propensity to weight gain (Reinehr 2010).

An in-frame deletion in NHSL1 at chr1:30,978,567-30,978,575 XM_038526147.1:c. 4011-4019gcAGGATCCGCg>gcg demonstrated an across-breed significance of P_height_= 0.008 and P_weight_= 0.004. An additional missense variant in NHSL1 at chr1:30,977,883C>T XP_038382074.1: p.A1313>T had a strong inter-variety p-value (P=0.0027) and across breed significance (P_height_= 1.41e^-06^ and P_weight_= 4.44e^-08^).

The gene *ARFGEF Family Member 3 (ARFGEF3)* is proposed as a systemic regulator of glucose homeostasis via the negative regulation of insulin granule biogenesis (Stelzer *et al.* 2016). A missense variant in this gene (chr1:30,793,904G>A, XP_038382065.1 p.V243>I) generates a substantive height difference (~10cm) across breeds 37cm for homozygous A/A versus 46-47cm for G/A or G/G (P_4BT_v_4MBT_ =0.0027; P_Across-breed___height_= 0.01, P _Across-breed___weight_= 0.74) (Table S3, Table S4). The modest across-breed P-value relative to the effect size is puzzling but may be influenced by the relative rarity of the “A” allele. The homozygous A/A genotype had a frequency of 0.007 in the Dog10K resource. A second missense variant in ARFGEF3 (chr1:30,820,591C>A) does not significantly influence height across breeds (P_Across-breed___height_= 0.41, P _Across-breed___weight_= 0.19).

#### Chromosome 3

The chr3 associated region: chr3:90,323,433-92,234,203, includes the previously described canine size gene *LCORL*. The *LCORL* gene transcript spans [chr3:91,736,787-91,867,349](https://genome.ucsc.edu/cgi-bin/hgTracks?hgsid=2130289718_VwAznPxhiyu0TFrA9BMFf7ISnamt&db=canFam4&position=chr3%3A91736787-91867349) on the UU_Cfam_GSD1.0 canine reference. The wider associated region contains 25 genotyping array variants with strong divergence between Bull Terrier breed varieties.

In the region of interest, the Dog10K VCF data shows 3,397 variants that are associated with breed-variety difference between BT and MBT with P<0.01. Of local variety associated variants, the most highly associated variant for height across breeds is chr3:91,734,656A>G (P_4BT_v_4MBT_ =0.007; P_Across-breed height_= 2.459e^-99^) and for weight was chr3: 91,706,639G>A (P_4BT_v_4MBT_ =0.007; P_Across-breed weight_= 9.762e^-85^). These were the strongest associations observed with across breed height and weight, respectively, across all regions of interest for the BT-MBT variety difference. The height variant affects a non-coding RNA (RLOC_0020773.1) annotated in the Uppsala University GSD1.0 gene annotations. The weight variant maps to a possible exon of the uncharacterized LOC119871273 (Fig.1, Fig. S2, Table S4). At both loci all four BT were homozygous for the reference allele while the MBT segregated the alternate allele. Across breeds, the variant chr3.g.91,734,656A>G additively decreases height by ~9 cm, and weight by 7.75 kg for each alternate (guanine) allele at the locus, while the variant chr3.g. 91,706,639G>A reduces height by 7.5cm, and weight by 9.9 kg for each additional alternate (adenine) allele.

#### Chromosome 10

There are no documented canine size genes in the region of interest on chr10 (chr10:22,419,486-27,856,291) (Raymond *et al.* 2022). The region of interest for Bull Terrier inter-variety difference includes 72 array variants (Table 1) and is extremely gene dense. The maximum observed association with breed variety is P_69MBT_v_33MBT_ =3.923e^-21^ (Table S2). Assessment of local variety-associated variants in the region of interest using the variant annotation integrator (VAI) (Hinrichs et al. 2016) suggested that a splice-site variant in the gene *Protein Kinase C And Casein Kinase Substrate In Neurons 2 (PACSIN*2) may be responsible for the observed association between BT and MBT (P_4BT_v_4MBT_=0.007), but there was no significant association with across breed height or weight in the broader data resource (P_Across-breed-height_=0.4655, P_Across-breed-weight_=0.1132 ) (Table S3). It is possible that this locus is identified due to population stratification, genetic drift, or selection for another trait.

#### Chromosome 18

The region of association encompassed chr18:30,893,366-37,177,480. The large (6 Mb) region contains no known or clear positional candidate genes for body size. Untranslated region and potential splice site mutations were associated with BT variety in *Intraflagellar Transport Associated Protein* (*IFTAP*) (P_4BT_v_4MBT_ =0.00042), *Low Density Lipoprotein Receptor Class A Domain Containing 3* (*LDRAD3*) (P_4BT_v_4MBT_ =0.0027) and *Peptidase Domain Containing Associated With Muscle* (*PAMR1*) (P_4BT_v_4MBT_ =0.0027) (Table S3).

On chromosome 18, variants in the gene *PAMR1* may related to a preference for increased muscularity in the BT standard (The Kennel Club 2009) which asks for maximum substance for frame size.

#### Chromosome 34

The associated region on chr34 (chr34:18,646,348-18,939,475) includes the first and second exons of the previously identified canine size gene *insulin like growth factor binding protein 2* (*IGF2BP2*) (Raymond *et al.* 2022). Our analysis identified four possible functional variants in the transcribed portion of IGF2BP2. Two of these occur at adjacent bases (chr34: 18,694,869-70). The MBT segregate a three bp deletion (chr34: 18,694,869-71ins>del). By refseq annotation, the deletion occurs in the 5’UTR of IGF2BP2, however, according to the canFam4 Dog: Uppsala University GSD1.0 gene annotations for transcripts (IGF2BP2.1) and (IGF2BP2.6) the mutation results in an in-frame deletion of a glutamine in exon1 of IGF2BP2 that is a part of a poly-glutamine tract in the gene. The across breed probability of association with height and weight was significant for the deletion variant chr34: 18,694,869-71ins>del (P_4BT_v_4MBT_ =0.001946; P_Across-breed height_= 1.65e^-9^; P_Across-breed weight_= 1.79e^-8^). The predicted effect across breeds is for the deletion variant to reduce mean height and weight in a dominant/recessive manner: del/del 44cm, del/ins 44cm, ins/ins 50cm; and weight; del/del 17 kg, del/ins 18 kg, ins/ins 24kg (Table S4).

#### Overview and limitations

The populations accessed for this publication come from two different studies. It is well known that strong genomic differences can occur through genetic drift or population founder effects that have occurred since the management separation of the two varieties. Thus, not all loci identified as separating the BT and MBT are expected to be related to the phenotype of interest (which is body size). While body size is also understood to be a complex trait controlled by many loci in all species, it has been shown in dogs that major effects on complex traits can be controlled by relatively few loci that have been subject to active artificial selection by humans (Rimbault et al. 2013).

The Dog10K data resource is shared in the form of a variant call file (VCF) (Meadows et al. 2023). These files report differences from the reference genome assembly in smaller variants (typically < 100bp). The observations of variants are limited by the sequencing technology and alignment strategies employed and these are heavily weighted towards SNP and smaller insertion-deletion variants from short-read sequencing technologies. Methods are not readily available at this time for recording larger structural variants in VCF resources and so it is possible that structural variants may play a role in gene regulation of size genes. VCF files can also miss true variants occurring in low quality sequencing data. Therefore, variant reporting in this study may not be exhaustive and it is possible that the true functional variants have been missed.

#### Acknowledgements

I would like to acknowledge the undergraduate students Patrick Raymond and Caitlin Venerussi who contributed to earlier versions of this analysis.

1. Hinrichs A.S., Raney B.J., Speir M.L., Rhead B., Casper J., Karolchik D., Kuhn R.M., Rosenbloom K.R., Zweig A.S., Haussler D. & Kent W.J. (2016) UCSC Data Integrator and Variant Annotation Integrator. Bioinformatics 32, 1430-2.

2. Meadows J.R.S., Kidd J.M., Wang G.D., Parker H.G., Schall P.Z., Bianchi M., Christmas M.J., Bougiouri K., Buckley R.M., Hitte C., Nguyen A.K., Wang C., Jagannathan V., Niskanen J.E., Frantz L.A.F., Arumilli M., Hundi S., Lindblad-Toh K., Ginja C., Agustina K.K., Andre C., Boyko A.R., Davis B.W., Drogemuller M., Feng X.Y., Gkagkavouzis K., Iliopoulos G., Harris A.C., Hytonen M.K., Kalthoff D.C., Liu Y.H., Lymberakis P., Poulakakis N., Pires A.E., Racimo F., Ramos-Almodovar F., Savolainen P., Venetsani S., Tammen I., Triantafyllidis A., vonHoldt B., Wayne R.K., Larson G., Nicholas F.W., Lohi H., Leeb T., Zhang Y.P. & Ostrander E.A. (2023) Genome sequencing of 2000 canids by the Dog10K consortium advances the understanding of demography, genome function and architecture. Genome Biol 24, 187.

3. Raymond P.W., Velie B.D. & Wade C.M. (2022) Forensic DNA phenotyping: Canis familiaris breed classification and skeletal phenotype prediction using functionally significant skeletal SNPs and indels. Anim Genet 53, 247-63.

4. Reinehr T. (2010) Obesity and thyroid function. Mol Cell Endocrinol 316, 165-71.

5. Rimbault M., Beale H.C., Schoenebeck J.J., Hoopes B.C., Allen J.J., Kilroy-Glynn P., Wayne R.K., Sutter N.B. & Ostrander E.A. (2013) Derived variants at six genes explain nearly half of size reduction in dog breeds. Genome Res 23, 1985-95.

6. Stelzer G., Rosen N., Plaschkes I., Zimmerman S., Twik M., Fishilevich S., Stein T.I., Nudel R., Lieder I., Mazor Y., Kaplan S., Dahary D., Warshawsky D., Guan-Golan Y., Kohn A., Rappaport N., Safran M. & Lancet D. (2016) The GeneCards Suite: From Gene Data Mining to Disease Genome Sequence Analyses. Curr Protoc Bioinformatics 54, 1 30 1-1 3.

7. The Kennel Club (2009) Bull Terrier Breed Standard. URL https://www.thekennelclub.org.uk/breed-standards/terrier/bull-terrier/.
