# Supplementary figures and images for: Insights into the genetics of body size in the Bull Terrier
Source: Anim Genet. 2025 Jan 28;56(1):e70000. doi: 10.1111/age.70000 (PMC11773297; doi:10.1111/age.70000)

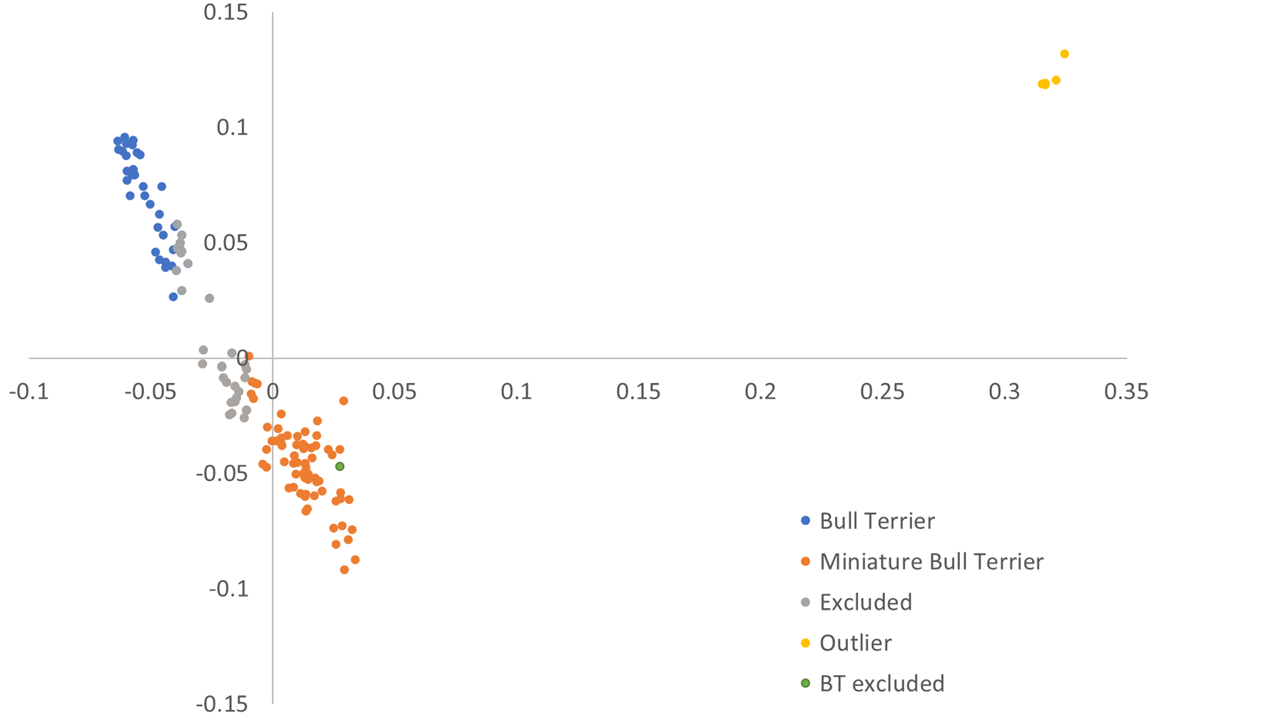

Supplement: Supplementary file 3 — Figure S1. [file AGE-56-0-s002.tif]

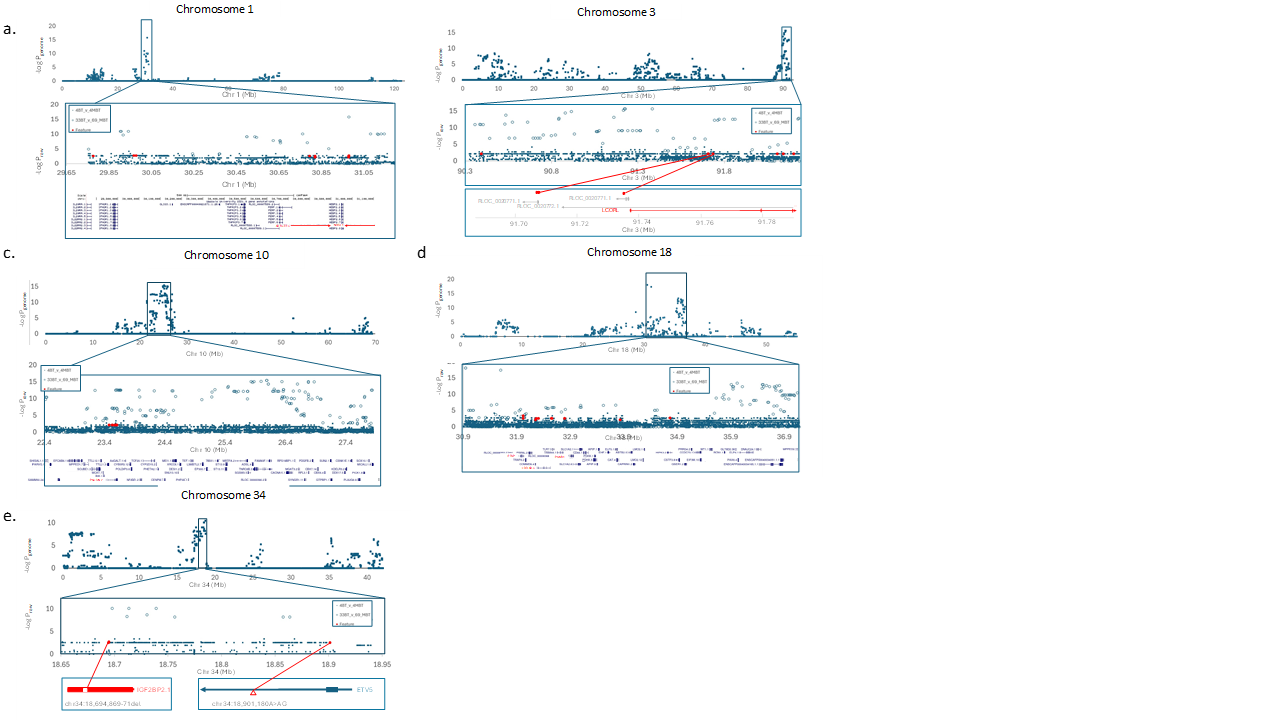

Supplement: Supplementary file 4 — Figure S2. [file AGE-56-0-s001.tif]
